# Autonomous Intersection Management: Optimal Trajectories and Efficient Scheduling

**DOI:** 10.3390/s23031509

**Published:** 2023-01-29

**Authors:** Abdeljalil Abbas-Turki, Yazan Mualla, Nicolas Gaud, Davide Calvaresi, Wendan Du, Alexandre Lombard, Mahjoub Dridi, Abder Koukam

**Affiliations:** 1 CIAD UMR 7533, Univ. Bourgogne Franche-Comté, UTBM, F-90010 Belfort, France; 2 Institute of Information Systems (IIG), University of Applied Sciences and Arts Western Switzerland (HES-SO), 3960 Sierre, Switzerland

**Keywords:** autonomous vehicle, cooperative intelligent transport systems (C-ITS), cooperative intersection management, virtual platooning, scheduling

## Abstract

Intersections are at the core of congestion in urban areas. After the end of the Second World War, the problem of intersection management has benefited from a growing body of advances to address the optimization of the traffic lights’ phase splits, timing, and offset. These contributions have significantly improved traffic safety and efficiency in urban areas. However, with the growth of transportation demand and motorization, traffic lights show their limits. At the end of the 1990s, the perspective of autonomous and connected driving systems motivated researchers to introduce a paradigm shift for controlling intersections. This new paradigm is well known today as autonomous intersection management (AIM). It harnesses the self-organization ability of future vehicles to provide more accurate control approaches that use the smallest available time window to reach unprecedented traffic performances. This is achieved by optimizing two main points of the interaction of connected and autonomous vehicles at intersections: the motion control of vehicles and the schedule of their accesses. Considering the great potential of AIM and the complexity of the problem, the proposed approaches are very different, starting from various assumptions. With the increasing popularity of AIM, this paper provides readers with a comprehensive vision of noticeable advances toward enhancing traffic efficiency. It shows that it is possible to tailor vehicles’ speed and schedule according to the traffic demand by using distributed particle swarm optimization. Moreover, it brings the most relevant contributions in the light of traffic engineering, where flow–speed diagrams are used to measure the impact of the proposed optimizations. Finally, this paper presents the current challenging issues to be addressed.

## 1. Introduction

Congestion is one of the critical concerns of this century. It generates energy consumption, pollution, delay, and stress. As the transportation demand grows, these problems dramatically increase. In urban areas, congestion is observed at intersections, where conflicting vehicles meet to share common road spaces. Therefore, intersection management has taken a big part in research works. From the end of the Second World War and even before [1], several papers contributed to improving traffic management at intersections, using traffic lights [2].

Traffic lights allow a higher average speed and improve the safety and throughput of the nodes of the traffic network in urban areas. Many technologies were introduced to control traffic. First, sensors (e.g., magnetic loops) were added to measure the traffic. Second, a control loop was implemented to provide adaptive traffic lights. Green and red times are adjusted to leverage the traffic conditions, using different techniques of forecasting, and optimization [3,4,5,6].

The recent progress in connected and autonomous vehicles (CAV) brings a new way of managing traffic in intersections. First, these vehicles communicate with the surrounding environment. They can transmit their states: their origin–destination, position, and speed. In turn, they get the right of way that fits their current state. Second, they autonomously control their motion to adjust their speed according to the received traffic sign, so they avoid useless deceleration–acceleration. Both communication ability and driving automation allow these vehicles to organize themselves at intersections autonomously. This self-organization capability, at the intersection, was the subject of an active research community for more than two decades. This new approach for managing intersections has several names in the literature, such as autonomous intersection management (AIM) [7], cooperative intersection management [8], an unsignalized intersection [9], and so on. For the rest of this work, AIM will be used to designate an intersection of CAVs exclusively, where driving automation and connectivity are harnessed for improving intersection performance.

With this paradigm shift toward more accurate traffic control, a new traffic management foundation is needed. Several objectives can be considered, such as energy consumption minimization, throughput maximization, and delay minimization. Two main decision-making problems are raised by AIM. The first one is the *scheduling* that determines which vehicle goes first and which one goes after, and so on. Scheduling is a combinatorial optimization problem. The second problem is the *motion control* that computes the speed profile of vehicles to make them safely and efficiently exit the intersections. The solutions to this problem are based on control theories, such as model predictive control (MPC) and Pontryagin’s maximum principle.

Both the aforementioned problems are dependent. First, the optimal trajectory depends on the vehicle’s rank to make the vehicle efficiently exit at a given time. Second, the optimal sequence depends on the time when the vehicle is physically able to clear the conflicting spaces. As a result, the raised problem is the optimal control of a hybrid system where both the vehicle’s rank (combinatorial optimization) and speed (control theory) need to be computed. In addition to the combinatorial explosion of discrete states, the traffic dynamic and real-time constraints make AIM’s optimization problems highly complex. For instance, each incoming new vehicle questions the already-found optimal solution. Several adapted models and techniques were suggested in the literature to get over the complexity. These have led to various approaches that need to be categorized within a comprehensive view of AIM.

This paper aims first to provide a clear vision of the tremendous contributions in the field of AIM. The provided vision allows a better understanding of the growing body of advances in the field and the easy identification of the following challenges that must be overcome. Second, this paper introduces an effective solution to the problem of the simultaneous optimization of speed and sequence. Based on the experiments and traffic theory, this paper measures the significance of gains obtained at an isolated intersection. Finally, it discusses the future challenges of AIM.

## 2. Autonomous Intersection Management: Preliminaries

**Figure 1:**
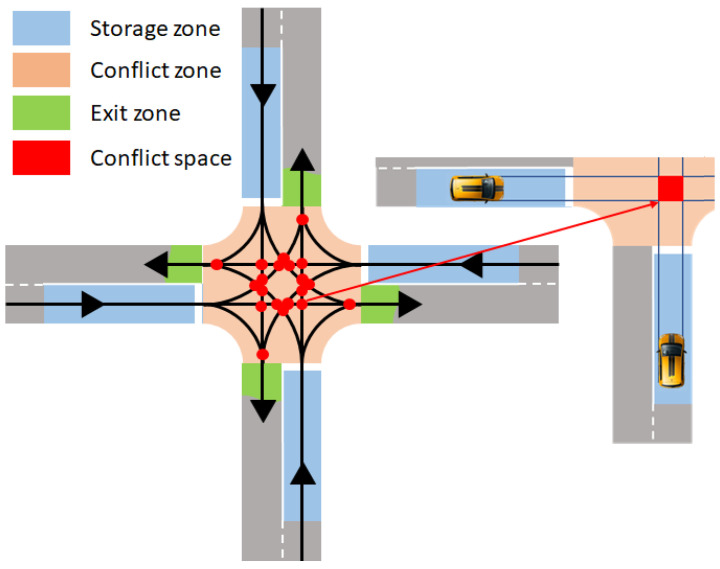
Intersection functional zones.

An isolated intersection may consist of several diagrams relating to the geometry of the infrastructure and the crossing rules. However, in each intersection, there are three functional zones (see Figure 1):

- Conflict zone: In this zone, the movement (origin–destination) trajectories intersect. In other words, it is the critical resource shared by all vehicles crossing the intersection, with a high potential risk of collision.
- Storage zone: It is located upstream of the conflict zone. It is the road before crossing the intersection.
- Exit zone: Downstream of the conflict zone. It allows the relief of the conflict zone.

The conflict zone of any intersection includes all conflict spaces (Conflict spaces are generally called conflict points. Indeed, it is a space that results from the intersection of two stripes as wide as the cars.). Each space results from the conjunction of at least two trajectories with a non-zero angle. For safety reasons, two vehicles from different lanes should not simultaneously access the conflict space.

Currently, traffic lights are the most advanced way to control access to conflict spaces explicitly. Traffic lights are installed at the end of the storage zone to show the color to vehicles before getting into the conflict zone. Traffic lights eliminate the major conflict spaces through a time-split mechanism—the so-called phases. The remaining conflict spaces in the same phase find a solution in the driver’s manual, such as a priority to the right for the turning left movement. The most advanced traffic lights are connected to vehicles in Intelligent Transport Systems (ITS). They are equipped with an ITS RoadSide Unit (RSU) for providing the Green Light Optimal Speed Advisory (GLOSA) service to the vehicles, equipped with an ITS OnBoard Unit (OBU). The service allows the connected vehicle to reach the green with an appropriate speed profile [10,11,12], for improving the riding comfort [13], saving time [14], and energy [15].

These gains obtained through the GLOSA are extended by the AIM. In addition to the speed control, the sequence computation eliminates all conflicts for each vehicle trajectory. So, there are no hard-coded phases. Instead, the CAV negotiates the right of way to pass through the conflict zone safely. With this in mind, AIM is an exclusively CAV traffic management at a single intersection that fulfills all the following features together:

- The accesses to the conflict zone are ordered (i.e., a sequence) according to the requests received from the CAVs via wireless communication.
- Each vehicle individually receives its own right of way concerning the sequence.
- Each CAV respects the received right of way by performing the suitable longitudinal control.
- Each vehicle instantaneously participates in the decision-making process by requesting the right of way and/or by communicating its current state.

This definition offers a wide range of possibilities for AIM implementation. Several AIM concepts are proposed in the literature. These concepts depend on the formulation of the optimization problem, its simplification, and the used algorithms and process to obtain solutions. For example, a note from the AIM definition that CAVs must both negotiate their schedule and adjust their speed. Which of these two problems (scheduling and trajectory optimization) deserves more attention? Which one should be addressed first? Similar questions arise from the perspective of problem allocation and distribution. Who performs the computation? If each CAV performs its own computation, how does the set of CAVs reach a consensus? The remainder of this paper aims at classifying the different concepts according to the choice made by the authors.

## 3. Autonomous Intersection Management: Review

To introduce the AIM literature, we use the following classification items:

- Protocol: It refers to how two conflicting CAVs share the common space to plan their successive passages. This defines the longitudinal control problem of the second vehicle to avoid a collision with the former one.
- Policy: It defines how the passage sequence is negotiated between the CAVs under real-time constraints.
- Architecture: It refers to the level of involvement of CAVs in the decision-making process. The level varies based not only on the problem statement but also on the assumed safety level.

### 3.1. Protocols: Cruise Control

**Figure 2:**
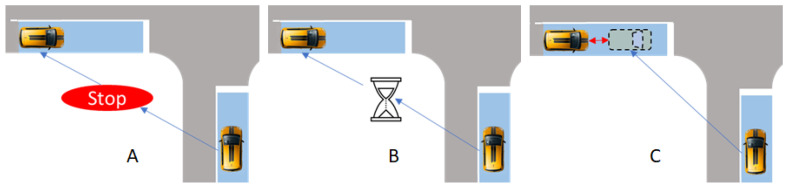
Protocols of AIM: (**A**)—Stop and Go, (**B**)—Reservation, (**C**)—Virtual Platoon.

Many protocols have been proposed for negotiating the successive passages of two CAVs. The most important protocols in the literature are as follows (See Figure 2):

- Stop and Go: The second CAV receives a stop sign until the first one leaves the conflict zone.
- Reservation: The first CAV sends the time when it leaves the conflict space, and the second one manages to get through the area later.
- Virtual platoon: The second CAV considers the first one as a virtual obstacle and adjusts its speed accordingly.

The cruise control strategy and the intersection efficiency depend strongly on the way the access to the conflicting space is managed, i.e., the protocol. Each protocol is detailed hereafter.

#### 3.1.1. Stop and Go

The Stop and Go is the first proposed protocol [16] in the literature. Each CAV has either a “Stop” or a “Go” sign. It is an extension of the ordinary traffic light, where the red is mapped to the “Stop” sign, and the green is mapped to the “Go” sign. The yellow sign is used only for sensor-based driving (on-sight mode) when a malfunction is detected [17,18]. Each CAV negotiates its own “Go” sign and obtains it individually. The cruise control design works as follows. The CAV considers the end of the storage as an obstacle when it has a “Stop” sign. When the CAV gets the “Go” sign, this obstacle is removed.

This protocol improves traffic light efficiency. First, it prevents vehicles from having a red sign on empty streets. Second, onboard signalization allows all non-conflicting movements. The challenge of the Stop and Go protocol is to select the best phase according to the current state of the vehicles. Hence, the Stop and Go protocol harnesses only the connectivity of the vehicles to improve the schedule. The driving automation capability of CAVs is not optimized.

Despite the simplicity of the protocol, the choice of the policy for getting the “Go” sign is not a trivial issue. In [19], the authors assume a V2V (vehicle-to-vehicle) communication with a “Go” sign by default. This sign turns into a “Stop” sign when other vehicles are discovered. A more conservative approach is proposed in [8,17,18,20,21,22]. The authors use an external server, V2I (vehicle-to-infrastructure) communication, to implement a default deny policy. When a CAV enters the storage zone, it has the “Stop” sign by default. Then, it needs to negotiate with the other CAVs through the intersection manager to get the “Go” sign. If there are no conflicting vehicles with the “Go” sign, the CAV is allowed to pass through the intersection. The permission is kept until the CAV exits. The CAV sends an exit request to the server to be removed from the list.

#### 3.1.2. Reservation

The reservation protocol was first introduced by Dresner and Stone in [23]. It uses the potential of driving automation. Rather than getting “Stop” or “Go” signs, the CAV gets the time when it is allowed to access the conflict zone and respects it. In the reservation protocol, the input control of the CAV is the time of entering and exiting the shared spaces. Hence, the reservation protocol requires performing two tasks: the time schedule of the CAV passages through the shared spaces and the longitudinal control to meet the due times.

The reservation time of the common space is computed according to the position and the speed of the CAV. This time is delayed if the common space is already booked. There are three kinds of common spaces considered in the literature. The most popular approach is splitting the conflict zone into several squares called tiles. The key rule is that one tile cannot be occupied by two CAVs simultaneously. The second approach books the conflict spaces [24,25]. The conflict space is a critical resource that can be used by only one CAV at a time. A more conservative approach makes CAVs reserve the whole conflict zone, as in [26]. In such a case, the time constraint to book the conflict zone depends on whether the preceding CAVs are conflicting.

To meet the reservation time, in [27], the cruise control is based on the computation of the time-velocity diagram with three pieces of a linear velocity function. More complex cruise control is studied in [28] by using Reinforcement Learning with discrete acceleration and speed values. In [26], the authors compute the optimal trajectory through Pontryagin’s maximum principle for minimizing the control effort. In [25], the authors use non-linear programming based on the infinitesimal method to minimize the energy consumption. A rolling strategy is used to overcome the problem of schedule feasibility during the CAV movement. Many papers are based on MPC to compute the speed profile, such as in [24,29,30,31,32,33]. Energy consumption is one of the most popular criteria to compute the optimal trajectory [34,35,36,37].

The reservation protocol raises two issues. First, deadlock may happen with the reservation of more than two potential conflict areas. In [38,39], the authors give a solution based on the elimination of cycles in the graph of priorities. Second, if a CAV does not respect the time, a collision will happen [40]. This safety issue is raised when the speed of a CAV in the booked common space is challenging to determine [39]. For instance, a CAV that meets congestion at the exit zone needs to slow down and delay its exit time. To overcome this problem, in [41], the authors add a high-speed segment upstream and downstream of the conflict zone. The conflict zone is crossed at the maximum speed of the CAV. However, this requires more lanes for considering the turning movements and more extended storage and exit zones. Other solutions were designed according to real tests. In [42], the proposed solution is based on buffer times and bigger tiles. In the tests described in [24], the authors add safety distances upstream and downstream of the conflict spaces. These safety distances allow the second CAV to come to a complete stop with emergency braking if the first CAV is still in the conflict space. Both solutions [24,42] increase the time between two passages of conflicting vehicles. In [43], the authors introduce an optimal safe state in the storage (position and speed) that the follower CAV respects to free the conflict spaces as soon as possible. With this state, the occupancy time is known, and the follower CAV can come to a complete stop if the preceding CAV does not respect its schedule.

#### 3.1.3. Virtual Platoon

This protocol was initially introduced by [44,45] and tested by [17] through real robots. After the contribution of [46], it is currently known as a “virtual platoon”. To overcome the safety issues raised by the reservation protocol, instead of booking a window of time, CAVs respect the sequence to control their speed. The sequence gives which CAV goes first, which one goes second, and so on. The sequence is obtained from an ordered list of presence (OLP) that is broadcast to all CAVs. Each CAV initially gives its origin–destination and periodically updates its current position and speed. In addition, each CAV identifies, from the OLP, the preceding CAVs and considers them as obstacles if they share the same conflict spaces.

In [44,45], the authors use the Gipps model [47,48] to control the simulated virtual platoon. In [49,50,51], the authors introduce the RT-CVC (Reaction Time-based Collaborative Velocity Control) [52] to consider communication and computation delays. More usual control techniques are adapted to the virtual platoon protocol. In [17], simulations of virtual platoons are based on an enhanced IDM (Intelligent Driver Model) [53] to compare a virtual platoon with the Stop and Go protocol. In [46,54,55], the authors use a linear controller concerning errors, whereas in [56], a sliding mode controller is tuned. Many simulations show that the virtual platoon is more efficient than the Stop and Go protocol. Moreover, they show that it is less efficient than reservation [56]. Nevertheless, simulation results need to be thoroughly studied according to safety constraints (see Section 3.1.2) and the scheduling algorithms, detailed in the next section (Section 3.2).

### 3.2. Scheduling

This protocol determines the way CAVs share the common spaces, either by yielding the way, by booking, or by following the preceding CAVs. It is obvious that the resulting cruise control strategy contributes to the improvement in the performance of the intersections. However, more is needed to thoroughly explore the potential of wireless communication, mainly when the traffic flow is high [57]. Scheduling is the other key to improving the intersection performance.

The scheduling problem of CAVs in the intersection is a combinatorial optimization problem. Some assumptions are formulated to model the problem. Most existing studies prohibit overtaking in the same lane of the storage zone. So, the order of arrival at a given lane is maintained when the CAVs exist. A more recent study [58] introduced overtaking. The author splits the scheduling optimization process into two stages of optimization. The first stage changes the order of CAVs, and the second one schedules access to the conflict areas. Mathematically, if overtaking is prohibited, the number of combinations is as follows:

(1) $$\frac{\left(\sum_{i=1}^{L}n_{i}\right)!}{\prod_{i=1}^{L}\left(n_{i}!\right)},$$

*L* and $n_{i}$ are the total number of lanes in the storage zones and the number of CAVs in the lane *i*, respectively. In Equation (1), the quotient is owed to the conservation of the order. Even with the quotient, Equation (1) shows a combination explosion.

The other assumption concerns the safety time between two successive passages through the conflict space. This time was first addressed by using traffic engineering theories to define the headway time. The time lost when the traffic light swaps from red to green [59,60] is used in the Stop and Go protocol [18,61]. Microscopic simulation [62] is used to determine the time constraint for scheduling the virtual platoon [45,49]. Finally, time constraints and/or the distance with the preceding CAV are defined in the reservation protocol. Note that there is no consensus on the safety constraints of the reservation protocol. This constraint strongly depends on the assumptions made about the ability of CAVs to respect their reservation time. Some authors assume a lower time between conflicting vehicles than the time between two following vehicles that move in the same lane [32]. This leads to significant performance gains. However, this assumption is highly questionable in practice [42].

Two approaches for scheduling the intersection are proposed in the literature:

- Exact and heuristic approaches: Depending on the arrival times of CAVs, their speeds, and positions, the intersection server calculates the optimal (near-optimal) sequence and time for getting into the common space.
- Policy: Considering that the intersection is dynamic and because of the real-time constraints, simple efficient rules are defined.

#### 3.2.1. Exact and Heuristic Approaches

To address the complexity issue, [61] introduces a dynamic programming algorithm that solves the $C_{max}$ scheduling problem of a cooperative intersection in a polynomial time and memory space according to the number of CAVs. However, the computation time and the memory space are exponentially increasing as the number of lanes increases. Another dynamic programming algorithm was suggested in [63]. It aims to minimize the number of nodes by putting together CAVs that can cross the conflict zone simultaneously, but there is no gain in terms of the computation complexity.

In [64], the authors propose a new traffic control strategy for an isolated intersection based on a Branch and Bound algorithm and a heuristic to evacuate the approaching vehicles as soon as possible. The structural properties of the problem are carefully investigated to simplify the search procedure of an optimal passing sequence. However, the calculation time is still high, especially considering numerous vehicles or several adjacent intersections.

From the scheduling theory, $C_{max}$ is the maximum completion time: the completion time of the last job (CAVs) in the system. Other more complex objective functions exist, such as the total weighted completion time, the average waiting time, or the maximum lateness [65,66,67,68]. In [69], a multiobjective optimization model for minimizing the delay, emission, and discomfort level is proposed. In [70], a vehicle–intersection coordination scheme (VICS) is proposed, which uses a risk score as the objective. To solve the problem with the other objectives, the authors resort to a mixed integer linear programming (MILP) model [71,72], either centralized [73] or decentralized [74]. For instance, to minimize the total travel time delay, [75] seeks the optimal vehicle scheduling at a multi-conflict area, considering heterogeneous vehicle headways and values of the time. The MILP model is proposed to provide the exact optimal solution to this problem. A similar approach is used in [25] to schedule the reservation of the potential collision area.

Only small instances of the proposed model can be solved by the existing commercial MILP solvers. The computational time increases exponentially as the number of vehicles and lanes increases. Hence, in practice, the use of optimal scheduling is limited to the following purposes:

- A comparison with the other scheduling approaches;
- Rolling horizon: The optimal schedule is computed for only a few CAVs.

The computation time and resource issues are also raised when the optimal solution is computed offline for comparison. Hence, the comparison with the exact algorithms can only be used for a short simulation period. This motivates many papers to introduce heuristics to obtain near-optimal solutions.

In [76], the authors give an analogy between the scheduling of a cooperative intersection and the well-known Traveling Salesman Problem (TSP) [77], where each CAV is considered as a city to be visited. This analogy invites the authors to use an ant colony system [78] to decide which CAV goes first, which one goes second, and so on. A more sophisticated ant colony system was introduced later in [79], for a decentralized negotiation between CAVs (see Section 3.3), where each CAV participates in the decision-making process. Genetic algorithms [80] are also used to schedule either a single intersection [81], priority vehicles [82], or a network of intersections [83,84]. In [83,84], groups of CAVs were formed to pass through the intersection together.

#### 3.2.2. Policies

A policy is a set of finite priority rules that are sufficient to solve a conflict between each pair of conflicting CAVs. There are many motivations to use policies rather than the classical scheduling algorithms presented previously. In [32], a policy is used to respect real-time constraints. Rules are also used very early to define simple agent’s behaviors, able to make the emergence of a global behavior that is close to the optimal [23]. The last motivation is that the system is dynamic. As shown in [18], each new arrival of a CAV in the storage zone can modify the previously found optimal solution. In other words, the global optimal solution is not necessarily the sum of the local optimal solutions [85].

To the best of our knowledge, it is possible to group the main policies in the literature into four families: First In First Served (FIFS), First Ready Out (FRO), Time To React (TTR), and Distributed Clearing Policy (DCP). The other policies are less common, such as the noticeable policy presented in [41,86,87,88]. The policy is based on game theory [89]. Instead of deciding who goes first, the system delays one conflicting CAV for each period (0.5 s).

**Figure 3:**
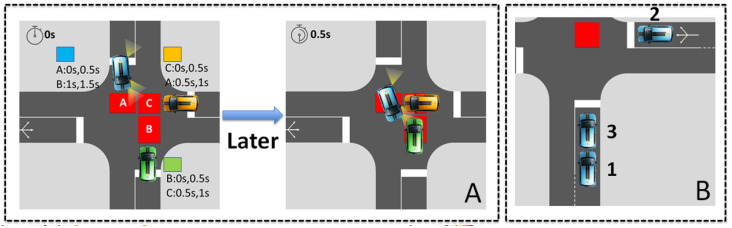
FIFS deadlock examples: (**A**) FIFS deadlock is the result of the reservation of tiles A, B, and C by blue, green, and amber CAVs; (**B**) FIFS deadlock is due to communication issues that raise the issue of order inconsistency (the number near CAV indicates the rank).

***First In First Served:*** FIFS is the most popular policy, especially for reservation and virtual platoon protocols. For instance, in the survey given in [90], the authors discuss only FIFS for the reservation protocol as an alternative to First Come First Served (FCFS). In the reservation protocol, it means that the first CAV who reserves the conflict zone, tiles, or conflict space has the right of way [23]. In the virtual platoon protocol, FIFS gives the right of way according to the order of arrival of CAVs [46]. Despite the simplicity of FIFS, a deadlock may happen because of communication problems and the resource model. In [38,39,91], the authors consider the deadlock that results from FIFS. Both former papers solve the circular wait due to the reservation of more than one conflict space (see Figure 3A). The last one considers the deadlock in the virtual platoon when messages are lost (see Figure 3B).

***First Ready Out:*** This policy means that when a pair of vehicles are in conflict, the CAV that can cross the intersection first will have the highest priority. The priority depends on the speeds and positions of both conflicting vehicles. It also depends on when the vehicles in front leave the intersection. Thus, in [92,93], the CAV has at least a lower priority than the one in front, in the same conflict zone. This allows us to avoid the deadlock situation presented in Figure 3b. In [94,95], vehicles follow an auction principle for reserving tiles. In a competing set of CAVs, the CAV that can clear the intersection earliest is the one that books the tiles first.

***Time To React:*** This policy was introduced for a distributed MPC in [29,31,33]. From [33], *“TTR is defined as the duration to the furthest point in time $t_{x}$ where the vehicle can still decelerate enough to come to a standstill in front of the intersection”*. Each CAV computes the TTR, which defines its priority. The CAV that has the lowest TTR goes first. In [29], the authors compare the TTR to FIFS and to the priority based on the remaining distance to the potential zone of collision. Both of the last policies were not feasible in terms of safety. However, this policy was only simulated for a few CAVs (six at most).

***Distributed Clearing Policies:*** A DCP was first introduced for the Stop and Go protocol. A DCP can be seen as an extension of the well-known vehicle-actuated traffic signal [96,97]. The lane that has the oldest CAV is chosen. The group is first formed by CAVs that belong to the lane, if they are close together. If a CAV that moves on the other lane can cross the intersection in parallel, it joins the group, and so on. It was mathematically proven that a DCP minimizes the queue length instantaneously if the headway time between two conflicting CAVs is at least two times bigger than the time space between two CAVs coming from the same lane [98]. Some works attempt to adapt the DCP to the virtual platoon protocol. In [17], two steps for forming groups in a real simple intersection of mini-robots are proposed. First, there is a small amount of time during which the new incoming robots negotiate together to form groups with the closest preceding robot in the same lane. Later, the robots adjust their speed together to avoid collisions. In [99], the approach is extended by splitting the storage zone into two zones. When a CAV arrives in the first zone, it joins the group called a bubble. Then, in the mid-zone, the bubbles are ranked by a branch and bound algorithm. In [100], safety rules are defined to allow a CAV to join the group. Similarly, in [101], the authors introduce a decentralized approach to form groups in the reservation protocol.

### 3.3. Architecture

Architecture refers to how CAVs communicate together to negotiate their right of way and get it. Architecture defines both the communication and computational hardware and software required to achieve the negotiations. Currently, there are several communication channels [102] that can be used for CAV negotiation in AIM, such as 5G (cellular network) [103,104] and G5 (Dedicated Short-Range Communications: DSRC) [105,106]. Telematics Control Units (TCU) [107,108] are used to carry the CAV connectivity with others (V2X: vehicle to others). Moreover, embedded high-performance computing system in vehicles is becoming more widespread [109,110] for vision, high-resolution display, and vehicle control. At the beginning of CIM studies, there were two opposing visions of the negotiation architecture, i.e., decentralized and centralized architecture.

#### 3.3.1. Decentralized Architecture

The first architecture was decentralized [7,16,111,112], where CAVs can self-organize their access to the conflict without the need for an external agent. This architecture avoids the use of external devices (ITS RSU) to manage the intersection. In this architecture, there is only V2V communication. However, the approach is questionable in terms of intersection safety. Because there is no intersection server, it is difficult to use a default deny rule. Each CAV that cannot communicate with others will react as it is alone. This results in collisions in the tests of the intersection of mini-robots presented by the authors of [113]. One solution is to oblige the CAV that is alone to slow down near the conflict zone to detect whether there are other CAVs. However, this solution may considerably lower the performance of the intersection.

#### 3.3.2. Centralized Architecture

The second architecture is centralized, where an intersection server communicates with CAVs to allow them to reach the conflict space safely. This architecture is entirely based on V2I communication. The task of the intersection is the optimization of the whole intersection, according to the used algorithm or policy.

This kind of architecture suits the use of optimization algorithms. The centralized approach was used for the scheduling optimization through algorithms [25,63,75,114] and heuristics [76,80]. From this point of view, the centralized architecture saves communication and calculation overhead compared to the decentralized one. The centralized architecture avoids that each CAV communicates its status to all surrounding CAVs and schedules the other CAVs’ access times. The server performs these tasks for the whole. A centralized architecture is also an opportunity to optimize together both the schedule and trajectory. However, except treating both problems in two separate steps as presented in [25], several papers witnessed that solving both problems together is currently unfeasible because of either the computation time [29,31] or convergence [32].

The centralized architecture alone is not sufficient to obtain a collision-free intersection. For instance, in [41], after the computation of the schedule, the intersection manager sends acceleration to only CAVs that need to decelerate or accelerate. CAVs that maintain their speed are not contacted by the server. Simply speaking, the CAV that does not receive a message from the server because of a communication issue will just continue its movement. However, the centralized architecture contributes to the safety enhancement through the default deny rule. The CAVs resort to a complete stop before the end of the storage zone if they cannot contact the server as discussed in [8,17,38,115].

### 3.4. Discussion

Based on this classification of the state of the art, the reader can easily observe the variety of concepts and the significant optimization potential of the AIM concept. However, although some choices may seem easy to implement, the evidence shows that each choice can potentially raise many problems related to the feasibility of AIM. AIM is indeed a real-time cyberphysical system that requires extensive study in terms of security and efficiency. The following two facts, i.e., the variety of concepts and feasibility, are discussed hereunder.

#### 3.4.1. Variety of Concepts: Hybridization

The contributions presented above clearly show the wide variety of configurations and optimizations offered by AIM. Various options are available to the designer. However, these options must not be perceived as antagonistic. In contrast, combining approaches is strongly recommended. For instance, one should not object V2V to V2I. The solution indeed lies in a hybrid architecture (V2X). This facilitates the consideration of intersection security through a common presence list distributed by the server and V2V to allow CAVs to negotiate their rank [100] in the list. This architecture makes it possible to benefit from the numerous distributed algorithms in the literature [116].

**Figure 4:**
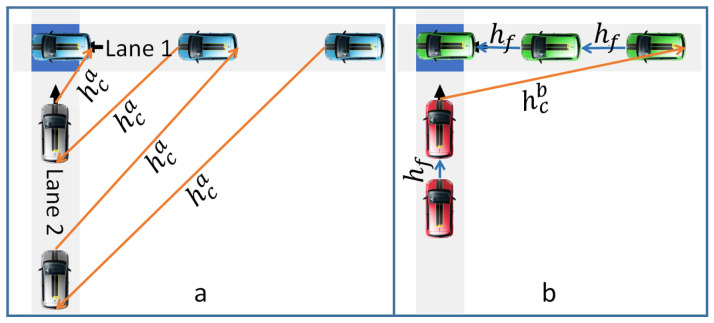
Example: (**a**) AIM based on speed adjustment and FIFS, (**b**) AIM based on sequence optimization and DCP.

This is also true for protocols and policies. The dynamic of the traffic requires different solutions that can be invoked based on the traffic context. The example presented in Figure 4 illustrates this statement concretely. This figure presents a simple intersection of five CAVs: three in lane 1 and two in lane 2. Two approaches are compared. In the first one (Figure 4a), the optimization is based on the speed adjustment so that two conflicting vehicles exit one after the other after three seconds ($h_{c}^{a}=3$ s). In the second approach (Figure 4b), the optimization is based on sequence optimization. Thus, the vehicles resort to a complete stop to let the flow of the other lane pass. Because of this stop, the time lost between the passage of two conflicting vehicles is six seconds ($h_{c}^{b}=6$ s). In both cases, two cars following each other in the same lane have a headway time of two seconds ($h_{f}=2$ s). If we focus on the exit time of the five present CAVs, both AIM strategies obtain the same result: 12 s. However, by removing the last CAV, the approach based on the speed adjustment (Figure 4a) is more efficient, whereas if a sixth CAV arrives at lane 2, then the sequence optimization approach (Figure 4b) is more efficient.

Despite its simplicity, the example must be seen in a more general way. The problem between speed adjustment and sequence optimization is not limited to this example. Speed adjustment reduces the opportunity for the scheduling task because AIM is a cyberphysical system. Physically, at a certain distance and speed, the vehicle is no longer able to brake to let another vehicle pass. The same applies to the optimization of the sequence. By adding a new CAV as a leader to the list, the follower will no longer be able to control its speed optimally. This example points to the value of each approach. The idea is not to eliminate either approach according to the traffic context. Conversely, this example advocates a smooth transition mode based on the CAV’s self-organizing ability to take the best of all possible approaches in every situation. This will be demonstrated by the system studied in the next section.

#### 3.4.2. Feasibility: Cyberphysical and Real-Time System

The other important remark that deserves to be pointed out is the complexity involved in implementing the AIM with real vehicles. The concept of AIM was introduced over twenty years ago, but the number of actual tests on vehicles remains very limited. Table 1 gives an overview of the tests. Some of these tests report some feasibility issues (see column collision). The number of actual tests is very low compared to the popularity of AIM and the number of contributions. This low number reflects the difficulty of reaching an entirely safe autonomous intersection. As underlined previously, many safety and deadlock problems are raised, which show that the implementation of AIM requires pervasive studies to cope with real-time and cyberphysical constraints.

Conceptual feasibility issues are often raised because of assumptions that are difficult to reach in practice. These assumptions can be summarized as follows. Many studies assume that the control of the vehicles is very accurate and that the vehicles make very small errors in estimating their future behavior. These assumptions are related to the vehicle positioning system [119] and the cruise control system. Despite the advances in the field of positioning systems, obtaining accurate positions remains a very active research area. In addition to GPS-RTK (Global Positioning System-Real-Time Kinematic) [120], SLAM (Simultaneous Localization And Mapping) [121], and inertial units [122], the authors propose ground position markers [17] or cameras that observe the scene to help CAVs [118]. Currently, new technologies of positioning allow foreseeing position accuracy unattainable in the past [123]. However, this does not totally solve the problems related to velocity control and the associated errors. The other strong assumption is the overestimation of the reliability of communication at regular short intervals. Indeed, AIM is simply not feasible with significant communication issues. However, it is possible to overcome common communication issues through a design that handles reasonable message delays and losses. Even though one can expect that networks will be more reliable and able to handle higher data rates [124], it is essential to consider fallback solutions. When they are not met, both strong assumptions lead to a deadlock and even collisions. Apart from the possibility of staking the AIM feasibility on the advances of future technologies, it was proven through tests that these problems could find solutions, such as using safety buffers to cover cruise control inaccuracy [24,42,118], deadlock prevention algorithms to overcome message loss [91], and so on. Moreover, this complexity invites more hybridization of the presented concepts. For example, it is quite conceivable to use the virtual platoon as a fallback solution to the reservation and the “Stop” and “Go” as a last resort.

## 4. Simulation Study: Cruise Control vs. Scheduling

This section compares the different concepts discussed earlier in terms of effectiveness. Four scheduling policies and three protocols are compared. Seizing the opportunity of the comparison, this section illustrates the hybridization advantage. The comparison is made through the simulation of a simple intersection. The metrics are based on the flow–speed curves.

### 4.1. Studied System

The studied system considers a typical four-leg intersection as the one presented in Figure 1. Note that there are no lanes dedicated to turning movements. The main objective of using such an elementary intersection is to assess the ability of AIM to save space dedicated to road infrastructure by improving the capacity to handle traffic. Indeed, this intersection has the advantage of gathering in the same lane CAVs that go straight, turn right, and turn left. The turning movements force vehicles to slow down, and the turning left generates more conflicts. The storage zone of each approach is 80 m in length and 3 m in width. The conflict zone is a square, with each side being 27 m long. CAVs are spawned 200 m away from the conflict zone. In each lane, 10% of CAVs turn left, 80% go straight, and 10% turn right. All spawned CAVs have the same characteristics. Their length is 4.4 m, and their width is 1.8 m. Their acceleration is bounded between 4 m/s${}^{2}$ and −4 m/s${}^{2}$ for comfort. The longitudinal control of the vehicles is managed so that they can respect the acceleration limits when a leading vehicle brakes at −6 m/s${}^{2}$ with a reaction time of 0.5 s [52]. The communication delay is bounded to 0.5 s, with a communication performance near 100 Megabits per second (Mbps). Each CAV is a passenger car equivalent [125,126]. The capacity of each lane is 0.5 pcps. The speed limit for CAVs that go straight is 50 km/h. When the CAV turns left or right, the speed is limited to not exceeding a lateral acceleration of 2 m/s${}^{2}$ (20 km/h and 16 km/h for turning right and left, respectively). Considering 90s of the cycle time of a two-phase traffic light and a lost time of 8 s, the capacity of the intersection is less than 0.77 pcps (see [127] for more details about the computation).

CAVs are generated with respect to the flow rate of each lane, given in pcu/s. The CAV generation follows Poisson’s distribution according to the flow rate [128]. This leads to a Markov model of the headway time [129]. The origin–destination is randomly distributed according to the probabilities of turning right, going straight, and turning left. The system uses both communication capabilities V2V and V2I. The infrastructure has an intersection manager that updates and broadcasts the OLP. The role of the intersection manager is to ensure the consistency of the list. Each CAV puts its origin–destination, current position, speed, the expected time for entering and exiting each conflict space of the intersection, and the version of the last received OLP.

The intersection manager receives request messages from the CAV and builds the OLP accordingly. It first behaves like a geo-networking filter to accept or refuse messages from CAVs. More precisely, the CAV position and direction in the messages will either make the intersection manager accept or refuse the CAV. The option is to drop the CAV from the OLP if it is in the exit zone. Second, it fills the list by default according to the dates of the CAV arrivals. The order of CAVs may change concerning the simulated scheduling policy. This task involves both CAVs and the intersection manager. The CAVs send a request to change their rank, and the intersection manager confirms whether the order changes. The rank is changed only if the intersection manager receives an acknowledgment message from all involved CAVs. The CAV that receives the OLP behaves differently according to the simulated protocol. Simulated policies and protocols are detailed hereafter.

### 4.2. Comparison of Protocols and Policies

**Figure 5:**
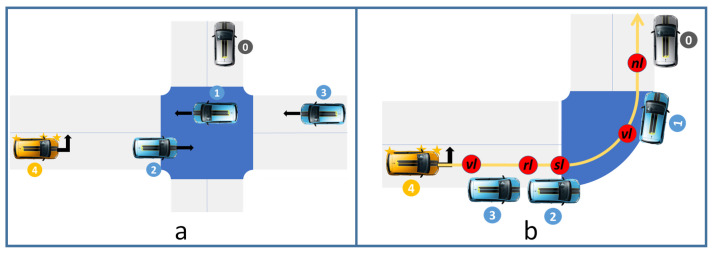
Virtual platoon: (**a**) Intersection state and CAV rank, (**b**) considered obstacles by CAV${}_{4}$. There are four kinds of considered obstacles: real leader rl (CAV${}_{2}$) detected by the sensor, virtual leaders vl (CAV${}_{1}$ and CAV${}_{3}$) that have intersecting trajectory with CAV${}_{4}$, next leader (CAV${}_{0}$) that is still in the exit zone of CAV${}_{4}$, and the stop line sl.

Table 2 summarizes the compared protocols and policies. There are seven scenarios that are compared. The presented architecture allows handling the three protocols, i.e., Stop and Go, virtual platoon, and reservation. For the Stop and Go protocol, the CAV has the right of way only if there is no conflicting CAV ranked before (at the top of the list). The simulated virtual platoon uses the Stop and Go protocol as a fallback. More precisely, each CAV considers the stop line as an obstacle if it is unable to keep a safe distance from virtual and next leaders, as presented in Figure 5. Finally, for the reservation protocol, the CAVs adjust their speed to keep a safe distance from all leaders [118]. An optimal safe state that minimizes the occupancy time of the conflict zone is computed as given in [43]. A safe state is the pair of position and speed that allows a CAV to come to a complete stop before the stop line, concerning its acceleration bounds. It is optimal if it allows the CAV to clear the conflict zone the soonest. The cruise control makes the CAV reach the optimal state, using the time-velocity diagram presented in [42]. The reservation time is computed accordingly. As the optimal safe state can be unreachable from a given initial state, the CAV that is unable to reach the optimal safe state (too near or too close to the conflict zone) is resorted to following the other CAVs according to the virtual platoon. The reservation time is computed according to either the optimal safe state (if feasible and no real leader), the minimum headway time with the real leader, or the speed of the vehicle and the maximum safety time space with virtual and next leaders (if the optimal state is not feasible).

**Figure 6:**
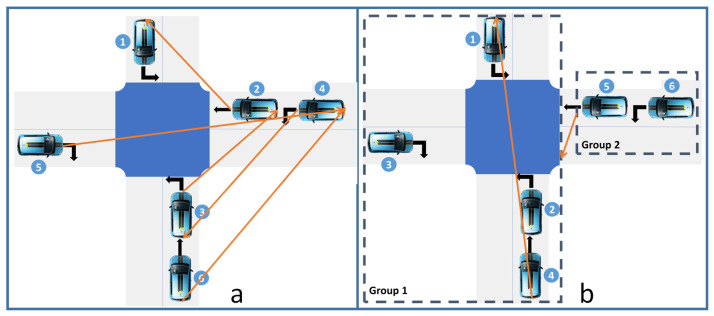
DCP: (**a**) Numbers give the initial rank and arrows represent the launched negotiation with virtual leaders, (**b**) The numbers give the rank after negotiation and arrows represent the set of virtual leaders.

Three policies are compared: (i) FIFS, where the initial rank given by the OLP is kept, unless for CAVs that can enter and exit the conflict spaces without conflict. This policy is simulated for the virtual platoon and reservation protocols. (ii) FRO which is only used simulated with the virtual platoon. In FRO, the OLP theoretically ranks the CAV according to the initial expected exit time. Because of cyberphysical constraints, this rank is adjusted not only to consider real leaders but also to avoid CAVs resorting to emergency braking. More precisely, a negotiation with all preceding conflicting CAVs is launched progressively, CAV per CAV, from the last to the top of the list. If the delay request is accepted, the rank changes in the OLP. For instance, in Figure 5, CAV${}_{4}$ begins the estimation of its exit time by considering the headway time with CAV${}_{2}$. If its exit time is shorter than the exit time of CAV${}_{3}$, it requests to be ranked before. CAV${}_{3}$ judges whether it accepts according to its proximity to the conflict zone. More precisely, it evaluates the required deceleration to come to a complete stop before the stop line. If the deceleration respects the constraint of −4 m/s${}^{2}$, CAV${}_{3}$ accepts. In such a case, CAV${}_{4}$ is ranked before CAV${}_{3}$, and so on. (iii) DCP, where CAVs try to form a group with the nearest opportunistic CAV met in the OLP with a temporal distance less than 2.1 s (see Figure 6). The opportunistic CAV is either the real leader or any other CAV without conflict. The group formation is also submitted to the progressive process of negotiation. A CAV can modify its rank only after negotiating its rank with other conflicting CAVs. However, the CAV can never be ranked before its real leader.

The DCP is simulated with the three protocols. Hybridization is introduced in the reservation protocol by modifying the acceptance condition of the rank swap. The positions of the optimal safe state equal 74 m and 75 m for straight and turning movements, respectively. In the reservation protocol, the rank swap is accepted only if the CAV can reach the optimal safe state. Hybridization relaxes this constraint by using the concept of a mobile synchronization point. The CAV *i* accepts the rank swap only if it can reach the synchronization point $sl_{i}$. Each CAV *i* generates its own $sl_{i}$ randomly, with 56 m$\leq sl_{i}\leq$ 80 m. The lower bound of $sl_{i}$ allows the CAV to reach its maximum speed when crossing the conflict zone, whereas the upper bound is the stop line. The mobile synchronization point needs to be adapted to the traffic state. If the traffic demand is low and there is no opportunistic change in the rank, the lower bound is a good solution, theoretically. Otherwise, when the traffic demand is high, it is better to form groups of CAVs (see the example in Section 3.4.1).

To make $sl_{i}$ fit the traffic state, we used a distributed approach inspired by the well-known particle swarm optimization (PSO). From the OLP, the CAV negotiates its rank with the CAVs in vl. According to its current $sl_{i}$, it considers only CAVs that are behind $sl_{i}$ to negotiate the rank. It compares the completion time of the new rank $C_{max}^{sl_{i}}$ to the completion of the preceding one $C_{max}^{old}$, considering only CAVs in vl. The fitness of the $sl_{i}$ is computed as follows.

(2) $$f\left(sl_{i}\right)=C_{max}^{old}-C_{max}^{sl_{i}}$$

If the result of the fitness of the current $sl_{i}$ is positive, it is kept as a local optimum $sl_{i}$. Whatever the result, after the negotiation, the CAV sends the computed $f(sl_{i})$ and $sl_{i}$ to the intersection manager. Among all the received fitness values during the step time *k*, it keeps the best value:

(3) $$sl^{*}\left(k\right)=argmax_{i}\left(f(sl_{i}\left(k\right))\right).$$

In other words, it keeps the $sl_{i}^{*}$ that gave the maximum gain of the completion time. The intersection manager computes the $sl_{IM}\left(k\right)$ that it will send later to the CAVs as follows:

(4) $$sl_{I}M\left(k\right)=\alpha sl^{*}\left(k\right)+\left(1-\alpha \right)sl_{IM}\left(k-1\right)$$

α=0.001 in the simulation (The value of α is computed empirically after several simulation runs). CAV *i* computes the new value of $sl_{i}$ for the step k+1 using the well-known particle’s position evolution of the PSO:

(5) $$sl_{i}\left(k+1\right)=sl_{i}\left(k\right)+V_{i}\left(k\right),$$

$V_{i}\left(k\right)$ is the particle velocity at step *k*. $V_{i}\left(1\right)$ is generated randomly when the CAV *i* is created. $-0.5\leq V_{i}\left(1\right)\leq 0.5$. For all other simulation steps, $V_{i}\left(k\right)$ is randomly computed as follows:

(6) $$V_{i}\left(k\right)=\beta V\left(k-1\right)+\phi \left(sl_{i}^{*}-sl_{i}\left(k\right)\right)+\psi \left(sl_{IM}\left(k\right)-sl_{i}\left(k\right)\right),$$

β, ϕ, and ψ are empirically set equal to $\frac{1}{3}$.

### 4.3. Simulation Results

**Figure 7:**
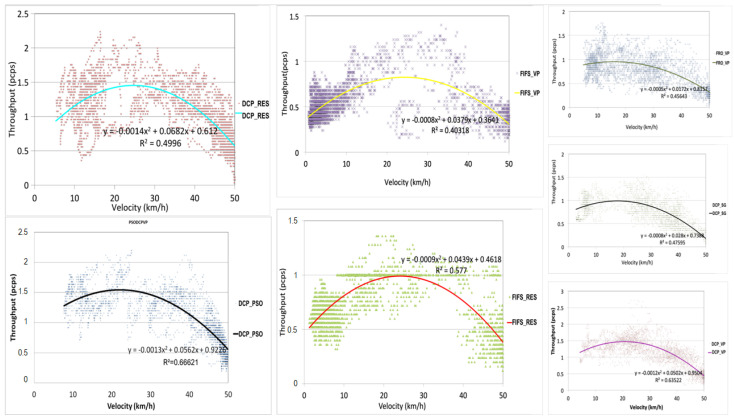
Flow–speed diagrams of the seven scenarios: simulation points, regression curve, and regression coefficient.

The seven scenarios of the AIM were compared using the flow–speed diagram. This diagram is built by drawing points of one minute of the simulation. In each minute of the simulation, point coordinates are computed from the number of CAVs that left the intersection (x-axis) and their average spent time from the storage zone to the exit zone (y-axis). Each policy is simulated with the traffic flows given in Table 3 during 10 min, six times. This provides, for each scenario, a set of 360 points. A quadratic polynomial regression is used to find the best fit and to determine the intersection capacity. The simulation results are given in Figure 7. The intersection capacities obtained by the quadratic polynomial regression are high because of the short simulation sampling time (1 min). This short sampling time allows the detection of simultaneous passages of more than two CAVs, which improves the comparability of the studied scenarios.

From this figure, one can analyze the distribution of the points in each scenario. The points on the right side are generated when the traffic is smooth. The points on the left side indicate traffic congestion. For instance, for the scenario FIFS_VP, most of the points are on the left side. In other words, congestion happened in most of the simulation runs. In the DCP_PSO, most of the simulation points are in smooth traffic. Because all the scenarios were submitted to the same traffic demands, it is simple to observe the one that can admit more CAVs (DCP_PSO). The other important analysis is related to points over 1 pcps. These points show the ability of the proposed approach to schedule more than two CAVs to pass together at the same time. This is possible through the formation of efficient groups, including turning movements. For instance, in Figure 6b, the first three CAVs are scheduled to cross the intersection together. From both standpoints, i.e., the concentration of points on the right side and points bigger than 1 pcps, it is easy to observe that the DCP_PSO gives the best results, whereas the FIFS_VP provides the worst results.

**Figure 8:**
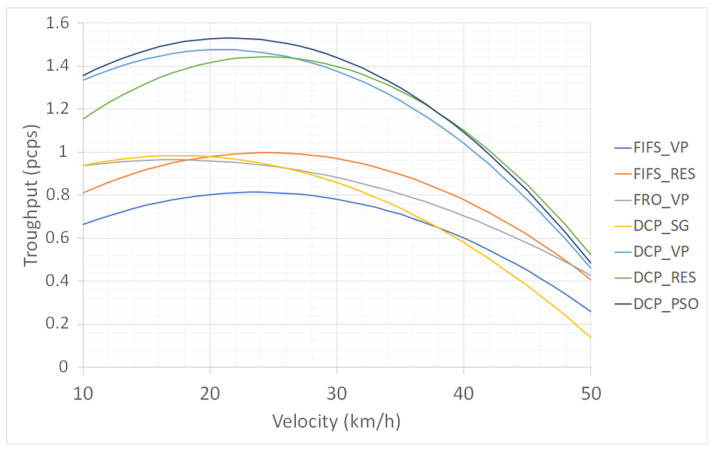
Comparison of the seven flow–speed regression curves.

Figure 8 compares the regression curves of the seven scenarios. The top (optimum) of each curve gives the average capacity of the intersection according to the related scenario. The four curves with a low capacity are the FIFS_VP, FRO_VP, DCP_SG, and FIFS_RES. Among the four scenarios, the DCP_SG is the one that exhibits the lower speeds in smooth traffic. This is an obvious result because the CAVs in this scenario are more likely to resort to a complete stop. However, the FIFS_VP is the scenario that has the lowest capacity to handle the traffic demand (near 0.8 pcps). The DCP_SG, FRO_VP, and FIFS_RES have approximately the same capacity that is close to 1 pcps. Although the cruise control in the DCP_SG is not optimized, the scheduling approach allows this scenario to reach a comparable capacity to FRO_VP and FIFS_RS. We can also easily observe the improvement brought by the cruise control with the FIFS policy. Indeed, the reservation has allowed for a significantly improved performance between the FIFS_VP and FIFS_RES. The same can be observed between the DCP_SG and the three other DCP scenarios. The protocol has significantly improved the performance of the intersection.

The three curves with higher capacities are the DCP_VP, DCP_RES, and DCP_PSO. The difference between the reservation and the virtual platoons are insignificant. The difference is observed when the traffic is low, with a slight advantage for the reservation that allows the CAVs to go faster. In congested traffic, the virtual platoon has a slight advantage. This is because the CAVs have more opportunities to form efficient groups. The PSO (DCP_PSO) approach outperforms both the reservation and virtual platooning, exploring the advantages of each approach according to the traffic state. However, due to the random generation of the synchronization points and low negotiation numbers when the traffic flow is low, the DCP_PSO takes less advantage of the reservation, as can be observed from the right side of the figure.

These simulation results show the potential for enhancing the traffic flow at the intersection. Both scheduling and cruise control contribute to significantly improving the intersection performance in terms of the speed and throughput. Although the physics of moving vehicles requires a choice between speed profile optimization and scheduling, the simulations showed that it is possible to leave this choice to the system to adapt to the situation by using techniques such as the distributed PSO. Neither FIFS with reservation nor the DCP with Stop and Go perform the best. The proposed PSO demonstrates that the safe distance can be maintained for a deceleration limited to −4 m/s${}^{2}$ while improving the throughput and speed at the intersection.

## 5. Discussion and Research Directions

The presented work shows the significant steps taken to improve future intersections. These intersections involve CAVs. Their ability to communicate and control their trajectory has allowed a paradigm shift. Scheduling algorithms and trajectory optimization techniques have replaced the calculations of phase splitting and cycle times to explore protocols for sharing conflict spaces. The majority of the works are concerned with either trajectory optimization or scheduling. The cyberphysical constraints of the system impose this choice. Nevertheless, more and more works involve both optimizations. In our example, we have shown that the two optimizations can be combined. They were adapted to the traffic state, giving more opportunity to either save the clearance time when the traffic is smooth or to form efficient groups of CAVs that cross the intersection together if the traffic is congested. We have also shown that combining the two optimization processes allows noticeable gains in the intersection performance, both in terms of the throughput and speed. These gains do not require additional lanes, which allows for saving urban space for other use.

The progress made in autonomous intersection optimization is noticeable, anticipating the future traffic demand to provide less congested cities and less paved urban spaces. However, the achievements of the AIM with real vehicles remain anecdotal. These achievements are limited to some feasibility trials and occasional demonstrations. This gap between the very promising performances and the realizations could be largely explained by the lack of equipment, such as precise positioning systems [130,131], safety monitoring systems for protection [132], and high-performance communication systems that meet the requirements of public use. For example, the issue of cybersecurity [133,134] will be legitimately raised and may result in additional communication costs [135] unanticipated by the current simulations. Nevertheless, these technological infrastructure shortcomings are related to the entire sector of autonomous and connected vehicles and are gradually being addressed to produce reliable solutions that will benefit the AIM.

Apart from the problems related to the maturing technologies and the lack of empirical tests using robots [136], virtual reality, and a mixed reality framework [137], the concepts related to AIM must also evolve to be feasible. Therefore, the three main research directions are the following:

***Vulnerable Road Users (VRUs):*** Because the AIM concept can be applied first in the context of autonomous shuttles in reserved areas, it is mandatory to consider VRUs. However, AIM focuses exclusively on optimizing the traffic of vehicles. As a result, CAVs are not even planned to come to a complete stop in case of conflicts (see the virtual platooning and reservation protocols in Section 3.1.3 and Section 3.1.2, respectively). This raises the question of sharing space with other road users, in particular the most vulnerable (VRUs). One option is to enable VRUs to take advantage of capacity gains. This is possible through Barnes’ dance concept [138]. The resulting capacity gains can be fully provided to the VRUs. The intersections will be managed in two phases. One phase is entirely devoted to vehicles. This phase will be used to the maximum through AIM. The other phase will be dedicated to VRUs, where pedestrians and cyclists can walk through the conflict zone in any direction they choose [139]. The design of such VRU phases needs to be studied in terms of user friendliness. Indeed, the VRU phase can also be launched using new CAVs–VRU interaction techniques based on a human–robot (or agent) interaction [140]. The base of the CAVs–VRU interaction is to predict what other users (cyclists, scooters, pedestrians, motorcyclists, and drivers, among others) intend to do next to make a proper movement decision [141]. CAVs will not only detect objects but also predict the behavior of other users and notify their intention to the rest of the road users [142]. For example, in [143], the authors explain that the interaction without non-verbal communication occurs through two stages: (i) *Communication of awareness* describes the entire process that must be carried out for the CAVs to detect and identify the VRUs. (ii) *Communication of intent* describes the capabilities of the CAVs to notify the VRUs of their next action (stopping or not stopping for the pedestrian). In [142], the authors consider that a third stage is necessary, called *broadcast communication*, which describes the different types of communication between the CAVs and the roadside units, infrastructures, VRUs, and other CAVs.

***Real-time multi-agent system:*** As mentioned in the previous sections, transiting vehicles and intersections can be modeled as distributed virtual entities (i.e., multi-agent systems—MAS [144]). However, conventional MAS models neglect strict timing constraints [145,146]. The notion of time (i.e., in the form of deadlines) is a prerogative of real-time systems (RTS). RTS are defined to be any information processing system which (i) has to respond to externally generated input stimuli within a finite, specified, and predictable response time; (ii) its correctness depends not only on the logical result but also on the time it was delivered; and (iii) a failure to respond *in* time is as bad as (or worse than) giving the wrong response [147]. Nevertheless, the real-time MAS (RT-MAS) modelization and approach presented by Calvaresi et al. [147,148] provide the right tools to satisfy both the need for distributed intelligence/flexibility and strict timing reliability. In particular, it models agents’ behaviors on real-time task models, defines the workload exchange, and defines/bounds a real-time reliable negotiation protocol. We envision employing the RT-MAS model with a hybrid architecture, enabling the rt-agents virtualizing the vehicles to interact and negotiate with each other and with the virtual rt-agent embodying the intersection. The rt-agents are expected to enact the reservation-based negotiation protocol [147]. The conflict zone (see Figure 1) can be modeled as the actual scheduler of the rt-agent intersection and the transiting vehicle as a sporadic task [149]—thus enabling us to use all the theories on real-time scheduling. The vehicles can interact with the intersection and among each other within the storage zone (see Figure 1). The underlying concept of priority can initially be modeled as FIFS, especially in the case that no competition affects the conflict zone. Conversely, as long as the selected vehicle has not yet occupied the conflict zone, a “preemption” can occur. It can be triggered by the intersection rt-agent if a vehicle with a higher priority (i.e., an ambulance in an emergency) approaches the storage zone or if a vehicle with a similar priority (again within the storage zone) asks for a “favor” due to hurry conditions. In such a case, if an agreement is found, a rescheduling takes place. Finally, the development of further awarding mechanisms, earned by renouncing its priority and spendable to “buy” priority, will be assessed.

***Multi-agent deep reinforcement learning:*** The problems raised by autonomous intersections reveal the limits of conventional problem-solving methods and techniques. Indeed, it is a hybrid dynamical system involving both discrete and continuous decisions, with a multi-criteria optimization problem (safety, capacity, energy, delays, etc.). To overcome the difficulty, the problem is either studied in a distributed manner, where each vehicle solves its own problem, or hierarchically in the form of distinct sub-problems, e.g., speed control, and scheduling. In any case, the formulation of the problem in a global way with one criterion, as presented in [26], does not find a direct global optimization technique. Moreover, the approaches used to solve the problem remain very demanding in terms of communication. This type of problem can be revived by multi-agent deep reinforcement learning [150]. It can even be an inspiration to design agents who can be trained to make consistent discrete and continuous decisions while saving communication overhead. Such agents would be trained to interact with other road users [151], such as VRUs or other ordinary vehicles.

## 6. Conclusions and Future Work

This paper presents the work that prepares the future management of intersections. The provided state of the art illustrates how CAVs can improve the current approaches to managing conflicts. Automated driving and connectivity will empower vehicles with the self-organization ability to use the conflict zone effectively. Behind this effectiveness lie two optimization problems: optimal cruise control and scheduling. The paper emphasizes that these two problems are different and involve distinct theories, models, and optimization techniques. They are often considered separately in other fields. This paper shows that AIM raises the challenge of dealing with both problems together to take full advantage of driving autonomy and connectivity. To this end, this paper simulates a set of protocols and policies and gives their speed–flow curves. A distributed PSO was used to adjust the speed profiles and sequences of CAVs with the observed traffic demand. As the demand increases, CAVs are more likely to slow down and yield the way, giving more chances to form efficient groups. The approach can be extended to many domains where scheduling and control problems are related.

This paper recalls the actual tests carried out in the literature. Despite their small number, they have the merit of, first, confronting the hypotheses with the realities of implementation and, second, testifying to the feasibility, even if only for a small number of vehicles. Based on the discussion of real-world tests and simulation results, this paper has presented a list of research directions that should be addressed to accelerate AIM’s progression and allow our cities to benefit from its advantages. Among these research directions, a thorough RT-MAS analysis coupled with actual tests will provide a significant leap forward in the field. This will prepare a safe and trustworthy AIM system that can be easily implemented in restricted areas, such as for personal rapid transit and Automated Guided Vehicles (AGVs) in industrial zones.

## Figures and Tables

**Table 1:** Real tests of AIM.

Ref.	Test	Veh.	No. of Veh.	AIM	Collision
[113]	8-shaped intersection	Mini-robots	6	Stop and Go, FIFS, Dec.	Yes
[39]	8-shaped intersection	CAV	2	Res., FIFS, Dec.	No
[42]	4-way virtual intersection	CAV	1	Res., FIFS, Cent.	Yes
[115]	4-way intersection	Mini-robots	4 (1 per lane)	Res., FIFS, Dec and Cent.	Yes
[8]	2-way intersection	CV	4	Stop and Go, FIFS and DCP, Cent.	No
[17,43]	2-way intersection	Mini-robots	3	Virt. Plat. and Res., FIFS and DCP, Cent.	No
[51]	8-shaped intersection	CAV	3	Vitr. Plat., FIFS, Cent.	No
[117]	4-way intersection	Mini-robots	4 (1 per lane)	Res., FIFS, Cent.	Yes
[118]	4-way intersection	Mini-robots	Unlimited	Virt. Plat., FIFS, Cent.	No
[24,30]	4-way intersection	CAV	3 (1 per lane)	Res., FIFS, Cent	No

**Table 2:** Compared protocols and policies.

	“Stop” and “Go”	Virtual Platoon	Reservation	Hybridization
FIFS	–	FIFS_VP	FIFS_RES	–
FRO	–	FRO_VP	–	–
DCP	DCP_SG	DCP_VP	DCP_RES	DCP_PSO

**Table 3:** Simulated flow.

Simulations	1	2	3	4	5	6
Flow per lane	0.1 upcs	0.18 upcs	0.2 upcs	0.25 upcs	0.28 upcs	0.3 upcs
Intersection demand	0.4 upcs	0.72 upcs	0.8 upcs	1 upcs	1.12 upcs	1.2 upcs
